# Photosensitization of a subcutaneous tumour by the natural anthraquinone parietin and blue light

**DOI:** 10.1038/s41598-021-03339-z

**Published:** 2021-12-10

**Authors:** María Laura Mugas, Gustavo Calvo, Juliana Marioni, Mariela Céspedes, Florencia Martinez, Silvia Vanzulli, Daniel Sáenz, Gabriela Di Venosa, Susana Nuñez Montoya, Adriana Casas

**Affiliations:** 1 Centro de Investigaciones sobre Porfirinas y Porfirias (CIPYP), CONICET and Hospital de Clínicas José de San Martín, Universidad de Buenos Aires, Córdoba 2351 1er subsuelo, CP1120AAF Buenos Aires, Argentina; 2 Departamento de Ciencias Farmacéuticas, Facultad de Ciencias Químicas, Universidad Nacional de Córdoba, Haya de la Torre y Medina Allende, Ciudad Universitaria, Córdoba, Argentina; 3 Consejo Nacional de Investigaciones Científicas y Técnicas (CONICET), Instituto Multidisciplinario de Biología Vegetal (IMBIV), Córdoba, Argentina; 4 Consejo Nacional de Investigaciones Científicas y Técnicas (CONICET), Córdoba, Argentina; 5 Instituto de Investigaciones Hematológicas, Academia Nacional de Medicina, Buenos Aires, Argentina

**Keywords:** Cancer, Cell biology, Drug discovery

## Abstract

Photodynamic therapy (PDT) is an anticancer treatment involving administration of a tumour-localizing photosensitizer, followed by activation by light of a suitable wavelength. In previous work, we showed that the natural anthraquinone (AQ) Parietin (PTN), was a promising photosensitizer for photodynamic therapy of leukemic cells in vitro. The present work aimed to analyze the photosensitizing ability of PTN in the mammary carcinoma LM2 cells in vitro and in vivo in a model of subcutaneously implanted tumours. Photodynamic therapy mediated by parietin (PTN-PDT) (PTN 30 µM, 1 h and 1.78 J/cm^2^ of blue light) impaired cell growth and migration of LM2 cells in vitro. PTN per se induced a significant decrease in cell migration, and it was even more marked after illumination (migration index was 0.65 for PTN and 0.30 for PTN-PDT, *p < 0.0001, ANOVA test followed by Tukey’s multiple comparisons test), suggesting that both PTN and PTN-PDT would be potential inhibitors of metastasis. Fluorescence microscopy observation indicated cytoplasmic localization of the AQ and no fluorescence at all was recorded in the nuclei. When PTN (1.96 mg) dissolved in dimethyl sulfoxide was topically applied on the skin of mice subcutaneously implanted with LM2 cells, PTN orange fluorescence was strongly noticed in the stratum corneum and also in the inner layers of the tumour up to approximately 5 mm. After illumination with 12.74 J/cm^2^ of blue light, one PDT dose at day 1, induced a significant tumour growth delay at day 3, which was not maintained in time. Therefore, we administered a second PTN-PDT boost on day 3. Under these conditions, the delay of tumour growth was 28% both on days 3 and 4 of the experiment (*p < 0.05 control vs. PTN-PDT, two-way ANOVA, followed by Sidak’s multiple comparisons test). Histology of tumours revealed massive tumour necrosis up to 4 mm of depth. Intriguingly, a superficial area of viable tumour in the 1 mm superficial area, and a quite conserved intact skin was evidenced. We hypothesize that this may be due to PTN aggregation in contact with the skin and tumour milieu of the most superficial tumour layers, thus avoiding its photochemical properties. On the other hand, normal skin treated with PTN-PDT exhibited slight histological changes. These preliminary findings encourage further studies of natural AQs administered in different vehicles, for topical treatment of cutaneous malignancies.

## Introduction

Photodynamic therapy (PDT) is an anticancer treatment involving the administration of a tumor-localizing photosensitizer (PS), followed by activation by light at its absorption wavelength. In the presence of molecular oxygen, PDT therapy results in a series of photochemical reactions mediated by reactive oxygen species (ROS) that cause irreversible photodamage to tumour tissues^1,2^.

Many hypotheses have been proposed to explain the selectivity of the PSs, including the presence of leaky and tortuous tumour blood vessels, the lack of proper lymphatic drainage known and in addition, some PSs bind preferentially to low-density lipoproteins, which can be overexpressed in tumours. Antitumour, antivascular and immune-related mechanisms of cell death have been found to be involved in PDT damage^3^.

In cancer dermatology, PDT mediated by 5-aminolevulinic acid, has a superior cosmetic outcome as compared to other forms of treatment such as curettage, freezing and other ablative techniques, and indications such as actinic keratoses, Bowen's disease and basal cell carcinomas have been approved many years ago^4,5^.

In addition to superficial tumours, PDT has been used for curative and palliative treatment of non-superficial tumours accessible by optic fibres. Either as neo-adjuvant therapy or in post-operative schemes, or in combination with radio- or chemo-therapy, many clinical trials involving PDT have been carried out^6^.

Even though PDT has been investigated for decades, only few PSs are approved for use in a clinical setting^6^. The most broadly used PSs employed in PDT of cancer are synthetic molecules. Among them, porphyrins, phthalocyanines and chlorophyll derivatives have been approved for clinical use; however, new PSs bearing higher selectivity and fewer dark toxicity are desirable.

Natural compounds are cheap and environmentally sustainable^7^ and moreover, herbal extracts have been employed to treat skin cancer among other malignancies^8^. Consequently, studies on natural PSs are an interesting area of research, as alternatives for the already approved PS for use in PDT of superficial cancers^7^.

Anthraquinones (AQs) are natural phenolic compounds whose chemical core consists of a planar and rigid anthracene ring with two keto groups at positions 9 and 10, and therefore, this structure allows the absorption of light of specific wavelengths. These features highlight their potential as natural PSs^9,10^. During the past few decades, both natural and synthetic AQs have been studied for their anticancer properties, and some of them such as doxorubicin, daunorubicin and mitoxantrone are nowadays the first-line treatment of many cancers^11^. The mechanism of action of AQs includes induction of aberrant cell metabolism**,** DNA damage, cell cycle arrest, apoptosis^12^, paraptosis^13^, autophagy^14^, radiosensitization^15^, decreased metastasis and invasion^16–18^. Doxorubicin and mitoxantrone are located in the cell nucleus, which is ascribed to the cationic alkylamino group responsible for its interaction with DNA^19^. Other natural AQs are mainly located in the cytoplasm, thus leading to a decreased mutagenic rate^20^.

The subcellular localization of the PSs has been proposed to highly influence the efficacy of PDT, since the light-induced damage takes place close to the PS site, due to the short half-life of reactive oxygen species provoking cell death^21^. It has been reported that AQs located in the cytoplasm induce apoptosis upon light irradiation^20^.

Previous reports described the photosensitizing activity of natural AQs and their in vitro cell killing effect mediated by singlet oxygen and superoxide anion radicals after illumination with UV-blue light^22,23^. In our previous work, we showed that the AQ Parietin (PTN), also named as Physcion, isolated from the lichen *Teoloschistes nodulifer* (Nyl.) Hillman (Telochistaceae) has a high singlet oxygen quantum yield^24^ and demonstrated to be a promising photosensitizer upon blue light illumination of cancer cells^25^, inducing a high percentage of apoptotic cells. However, there is limited or no knowledge about the selectivity of PTN for tumour tissue employing in vivo systems. Therefore, in this work, we carried out in vitro and in vivo studies to envisage the photodynamic action of PTN mediated by blue light (PTN-PDT) in superficially located cancers.

## Materials and methods

### Reagents

MTT (3-[4,5-dimethylthiazol-2-yl]-2,5-diphenyl-tetrazolium bromide) reagent and Crystal violet were obtained from Sigma Aldrich Corp (Merck KGaA, Darmstadt, Germany) and Canadian balsam from Biopack (Argentina). All reagents and solvents were purchased from commercial suppliers and used without further purification.

### PTN extraction and purification

**Figure 1:**
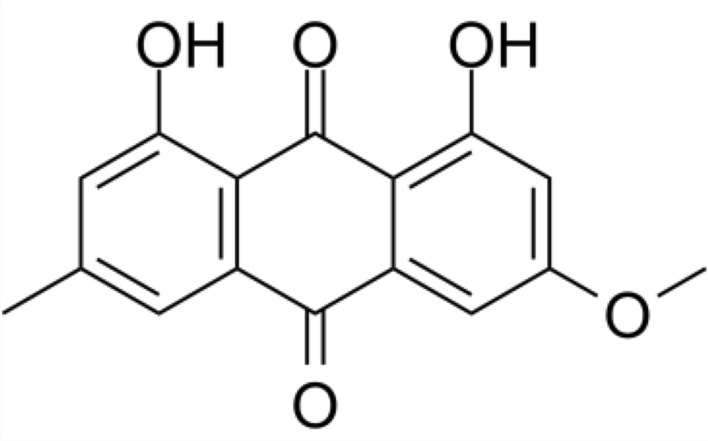
Structure of parietin (1,8-dihydroxy-3-methoxy-6-methyl-9,10-anthraquinone).

PTN (Fig. 1) was extracted and purified from the lichen *Teoloschistes nodulifer* (Nyl.) Hillman (Telochistaceae) as it was previously described^25^.

### PTN solutions

Stock solutions of PTN (46 mM in vivo and 5 mM in vitro) in DMSO were kept at − 70 °C until its use. The solutions employed in vitro were daily prepared by diluting the stock with RPMI medium (Gibco, USA), which contained 5% fetal bovine serum (FBS, Natocor, Argentina) and a concentration of DMSO ≤ 1%.

### Cell lines

LM2 murine mammary adenocarcinoma cells were kindly provided by Dr. Ana M. Eiján from the Instituto de Oncología Angel H. Roffo, Universidad de Buenos Aires, Argentina^26^. The cells were cultured in RPMI medium, supplemented with 5% FBS, 2 mM l-glutamine, 100 IU/ml penicillin, 100 mg/ml streptomycin and maintained at 37 °C in a 5% CO_2_ atmosphere. Cells were detached with 0.1% trypsin–EDTA and viable cells were counted using a hemocytometer. An amount of 7 × 10^4^ cells/ml cells were seeded in 24-well plates, incubated at 37 °C and were employed 48 h after plating.

### PTN-PDT treatment in vitro

LM2 cells were seeded in 24-well plates 2 days before PTN exposure. They were incubated in complete medium containing PTN at 30 μM for 1 h. Then, PTN was replaced by fresh medium and cells were irradiated with different light doses. After illumination, the cells were incubated for another 19 h, in order to evaluate the PDT effect. Controls of cells treated with PTN non-illuminated, and cells illuminated without prior PTN-treatment were included.

### MTT viability assay

The MTT assay^27^ was used to determine cell death after PTN-PDT and PTN dark toxicity. The absorbance of the resulting formazan was quantified at 560 nm in an Epoch microplate reader (BioTek, USA).

### Sub-cellular localization of PTN

The LM2 cells were seeded in coverslips and 48 h afterwards, they were treated with PTN 30 μM for 1 h. Then, the coverslips were washed with PBS and then inverted on slides with DABCO antifade, and fluorescence was observed in a BX51 Olympus microscope (Olympus, Japan) equipped with a 100 W mercury lamp, and the UV 330–385 nm excitation filter was employed. Photographs were documented with a digital camera (Olympus Q-color 5, Japan).

### Cell morphology after PTN-PDT

The cells were seeded on coverslips and treated with PTN-PDT as indicated above. Then, they were fixed with methanol and washed with PBS. After that, they were incubated with a crystal violet solution (1%) for 20 min and subsequently washed with distilled water. The dried coverslips were mounted on Canadian Balsam and cellular structures were observed under a light microscope. Controls of cells treated with PTN non-illuminated, and cells illuminated without prior PTN-treatment were included.

### Impact of PTN-PDT on cell migration

Cell migration of cells surviving PTN-PDT was determined employing a wound-healing assay^28^. The LM2 cells were seeded in 6-multiwell plates and allowed to grow until confluency, and afterwards, they were treated with PTN-PDT as indicated above. Immediately after PTN-PDT, cultured cells were gently wounded through the central axis with a sterile 200 µl tip, washed with PBS and refreshed with medium with serum. After overnight incubation (19 h) at 37 °C, the cells were fixed with formaldehyde, stained with crystal violet (1%) and photographed with an Olympus microscope with 4X objective and processed using image processing software (Fiji ImageJ 2.0.0-rc61/1.51n)^29^. Quantification of cell motility was performed by measuring the distance between the invading fronts of cells in 3 random selected microscopic fields. The degree of motility was expressed as a migration index $\left( {{\text{MI}} = \frac{{0 \;{\text{h }}\;{\text{wound}} - 19\;{\text{h}}\;{\text{wound}}}}{{0\;{\text{h}}\;{\text{wound}}}}} \right)$. Controls of PTN and Light were also included.

### Animals

Female BALB/c mice, 8 weeks old, weighing 20–25 g, were used. They were provided with food (Molinos Río de la Plata) and water ad libitum. A suspension of 1 × 10^6^ cells of the LM2 cell line was subcutaneously injected on the dorsal flank of female BALB/c mice. Experiments were performed at approximately day 8 after implantation. Tumours of the same uniform size were employed (0.5–0.8 cm diameter). The take rate of the tumours following transplantation was nearly 100%. Animals were divided into treatment and control groups by simple randomization (n = 14 per group). Mice were sacrificed if the tumour reached a size larger than 1 cm in diameter or if the tumour became ulcerated. We ensured that our protocols are refined to minimize the discomfort of the animals. Animals protocols were approved by the Argentinean Committee (CICUAL, School of Medicine, University of Buenos Aires) in full accord with the UK Guidelines for the Welfare of Animals in Experimental Neoplasia^30^. The reporting of in vivo experiments follows the recommendations in the ARRIVE guidelines.

### PTN topical administration to mice

Three volumes of 50 µl of a PTN solution (46 mM in DMSO) were subsequently applied on the skin overlying the tumour (SOT) of mice, after shaving the hair and rubbing with a smooth paintbrush for 3 periods of 5 min each in a time range of 1 h (total volume 150 µl, and 1.96 mg). The 46 mM concentration was chosen to perform the in vivo experiments since it is the maximum solubility of PTN in DMSO. Controls received DMSO topical application.

### Irradiation system

A bank of two blue compact fluorescent lamps (Sica 15 W model 914173, Argentina) was used. The spectrum of light has a maximum between 400 and 450 nm, which corresponds with the maximum absorption of a PTN solution in water and organic solvents^31^. The light source was located at a distance of 14 cm from the plates containing the cells (irradiated from below) and from the mice (irradiated from above). The fluence rates were measured employing a Field Master power meter and an LM3 HTP sensor (Coherent Inc., USA). The power density on the irradiation sites was 4.7 mW/cm^2^. Different light doses, which do not generate additional hyperthermic effects, were obtained varying the time exposure to the lamp.

### PTN-PDT treatment in vivo

PTN-PDT consisted of topical application of a PTN solution on SOT, and after 2 h, the mice were anaesthetized by intraperitoneal injection of 70 mg/kg ketamine hydrochloride and 6 mg/kg xylazine. Immediately, the tumours and normal skins were superficially illuminated with a light dose of 12.74 J/cm^2^ (45 min). PDT-PTN was performed twice with an interval of 1 day between treatments. After PDT, the animals were left to recover on a heating blanket.

### PTN penetration into the tumour

Two hours after topical application of PTN on SOT, mice were sacrificed and samples of their tumours with their adjacent SOTs were excised. Controls topically treated with DMSO were also performed.

For visualization of PTN fluorescence, SOT + tumour, samples were frozen and 15 µm thick cryosections were prepared. Both samples and sections were always handled under subdued lighting conditions. The penetration of PTN into the tumour was evaluated by fluorescence microscopy employing UV light excitation. A BX51 Olympus microscope (Olympus, Japan) equipped with a 100 W mercury lamp under these conditions: 330–385 nm excf, 400 nm dm, and 420 nm bf was employed using a 10 × magnification objective (BX51 Olympus microscope, Japan), and photographs were documented with a camera (Olympus Q-color 5, Japan).

### In vivo fluorescence spectroscopy after PTN application

The kinetics of PTN fluorescence was followed in vivo after topical application on the skin overlying the tumour of mice. A bifurcated optic fiber probe was coupled to a Perkin Elmer LS55 fluorescence spectrometer (Perkin Elmer, USA), and fluorescence from the tissue was detected at the probe tip. Excitation light at a wavelength of 460 nm was coupled into one arm of the bifurcated probe, enabling conduction of excitation to the skin surface and collection of the PTN fluorescence emission via the other arm to the spectrometer. The fiber tip was fitted with a rubber spacer that ensured a constant fixed distance of 7 mm between the fiber and the skin, providing optimal fluorescence signal collection. Fluorescence emission spectra were recorded at the application site of PTN as a function of time. In addition, fluorescence emission spectrum of a PTN solution in DMSO was measured to verify that the signal corresponds to PTN.

### Histological studies

One day after the second dose of PDT, mice were sacrificed and samples of tumours with their adjacent SOTs and normal skins were excised, extended, sliced, fixed in 10% buffered formalin, embedded in paraffin, sectioned, stained with hematoxylin and eosin, and examined by light microscopy under 4 ×, 10 × and 40 × magnifications (BX51 Olympus microscope, Japan), and photographs were documented with a digital camera (Olympus Q-color 5, Japan). The presence of tumour tissue, necrosis and other signals of tissue damage was evaluated. Epidermal and dermal damage, vascularity changes and the presence of lymphocytic infiltration were also investigated.

### Assessment of tumour response after PTN-PDT

The measurement of tumour size by caliper was employed to evaluate the delay of tumour growth induced by PTN-PDT. The longer (l) and shorter (w) perpendicular axes and height (h) of each tumour were determined daily with caliper since the period from the day of PDT (day 1) up to 1 day after the second PDT dose (day 4). Tumour volume was calculated using the formula l × w × h × 0.5, where 0.5 is a correction factor empirically determined^32^.

### Statistical analysis

The values of the wound healing assays were expressed as means values with standard deviations of at least three independent experiments. ANOVA test followed by Tukey’s multiple comparisons tests was performed to determine statistical significance between means.

To assess the tumour growth after PTN-PDT, two-way ANOVA, followed by Sidak's multiple comparisons test was used to establish the significance of differences between groups (controls vs*.* treated). Differences were considered statistically significant when p < 0.05.

## Results

**Figure 2:**
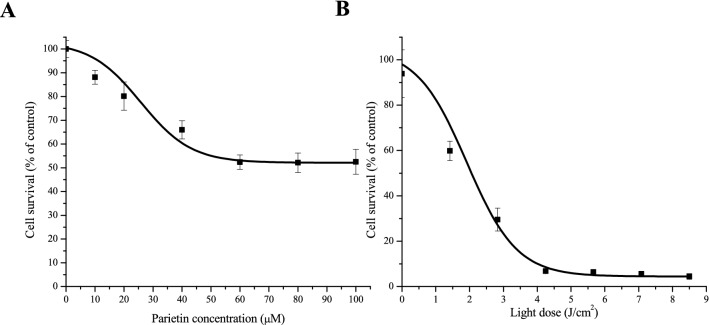
PTN toxicity in vitro on LM2 cells and response to PTN-PDT. (**A**) The LM2 cells were incubated with increasing concentrations of PTN for 1 h and afterwards, the medium was changed, and the cell damage was left to occur for a further 19 h period, simulating the PDT schedule. Cell viability was evaluated by the MTT assay, as the percentage of cells exposed to the vehicle. (**B**) Cells were incubated for 1 h with PTN at 30 µM and illuminated with different doses of blue light. Cell viability was evaluated after 19 h and expressed as the percentage of the PTN-treated non-illuminated controls.

Figure 2A shows that LM2 cells exposed 1 h to PTN induced up to 30% cell death at 30 µM. This was the concentration employed throughout this work since when PTN was employed at this or higher concentrations, it formed microscopically visible crystals (1% DMSO as co-solvent)^25^. When cells previously exposed to PTN were illuminated, a light dose of 1.78 J/cm^2^ was required to induce 50% of cell death. In addition, 4.5 J/cm^2^ induced the maximal cell death achieved that is 95% (Fig. 2B).

**Figure 3:**
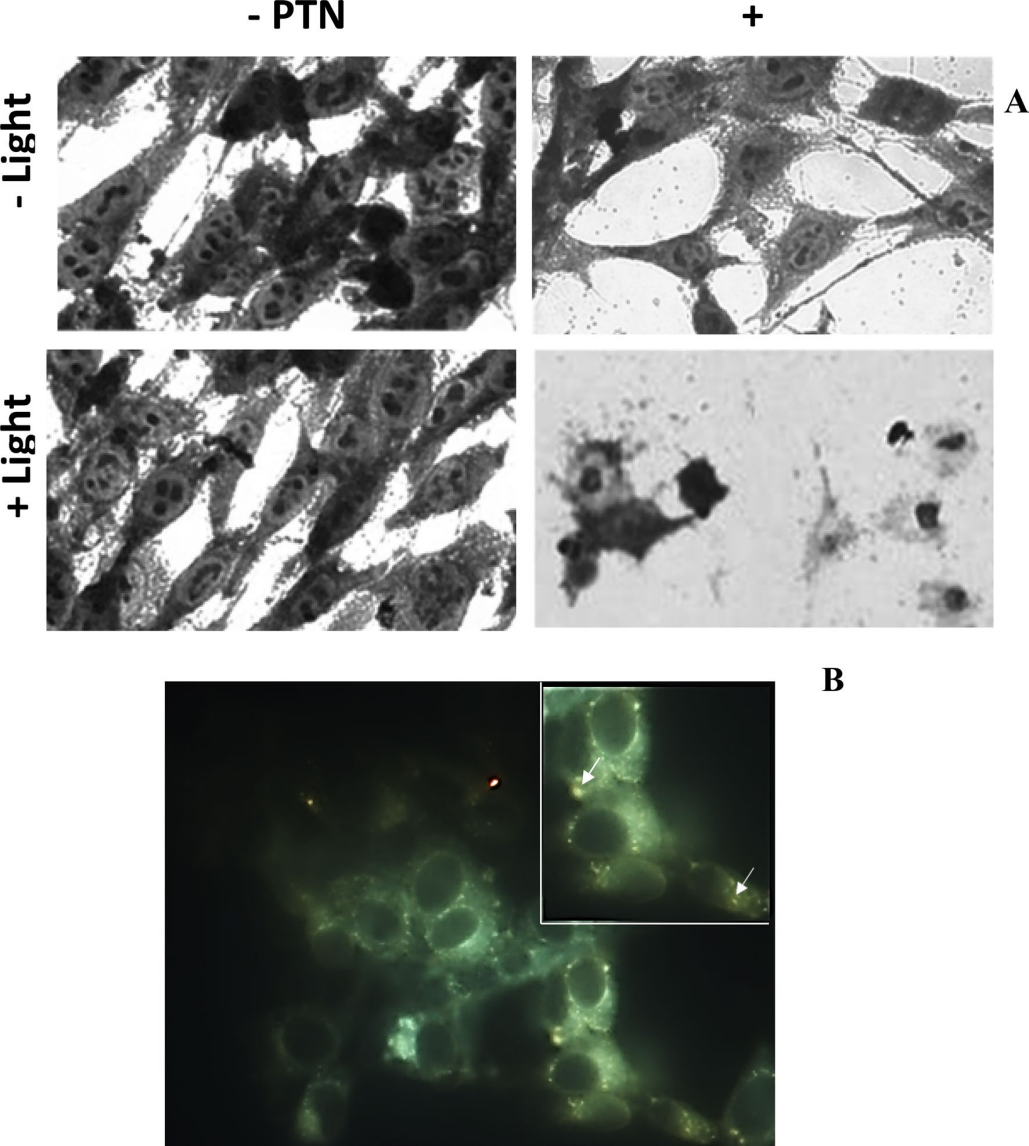
Morphological changes induced by PTN-PDT in LM2 cells and intracellular PTN localization. (**A**) LM2 cells were exposed 1 h to 30 µM PTN and immediately illuminated with 1.78 J/cm^2^. Cells were fixed after overnight incubation and stained by crystal violet for morphology observation. Micrographs were taken employing × 40 magnification. (**B**) LM2 cells were exposed 1 h to 30 µM PTN and fluorescence was documented at × 20 and × 40 (inset) magnification. Arrows indicate orange granules of PTN. Representative images are shown.

After blue light illumination of LM2 cells, PTN-PDT induced morphological changes such as massive cellular loss, condensed nuclei, and cytoplasmic vacuolization (Fig. 3A). Slight morphological changes such as cytoplasmic vacuolization were observed in some of the cells after PTN exposure without illumination.

Upon fluorescence microscopy inspection, PTN exhibited a diffuse orange-green fluorescence after excitation with UV light. A cytoplasmic localization pattern was observed, and it is noticeable that no fluorescence at all was recorded in the nuclei (Fig. 3B). Control cells incubated with the vehicle did not exhibit any significant fluorescence (data not shown).

**Figure 4:**
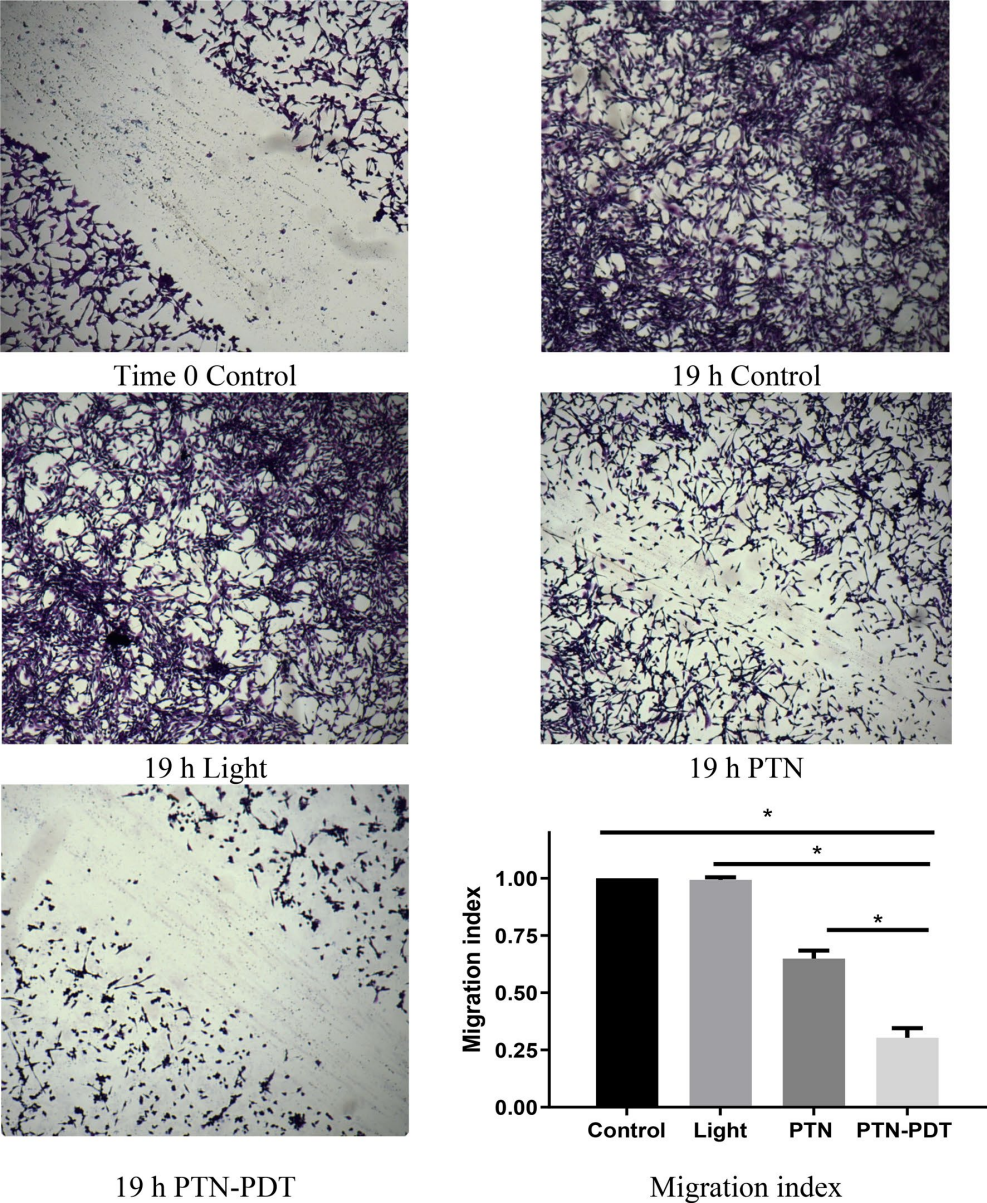
Impact of PTN-PDT on cell migration. LM2 cells were exposed 1 h to 30 µM PTN and immediately after 1.78 J/cm^2^ illuminations, they were wounded. Cell migration was documented after overnight incubation (19 h). Representative images processed using image processing software (Fiji ImageJ 2.0.0-rc61/1.51n) are shown: control cells at time 0 before migration (MI = 0), control cells treated with the vehicle at 19 h (MI = 1), illumination and PTN controls at 19 h and PTN-PDT at 19 h. Migration indexes were calculated. *p < 0.0001, ANOVA test followed by Tukey’s multiple comparisons test.

To assess the impact of PTN-PDT on the migration of the tumour cells, a wound-healing assay was performed (Fig. 4). The assay revealed that whereas PTN induces per se a significant decrease in tumour cell migration, the migration index was even more impaired after application of PDT (the migration index varied from 0.65 ± 0.04 for PTN treatment to 0.30 ± 0.03 for PTN-PDT), suggesting that both PTN and PTN-PDT would be potential inhibitors of metastasis.

**Figure 5:**
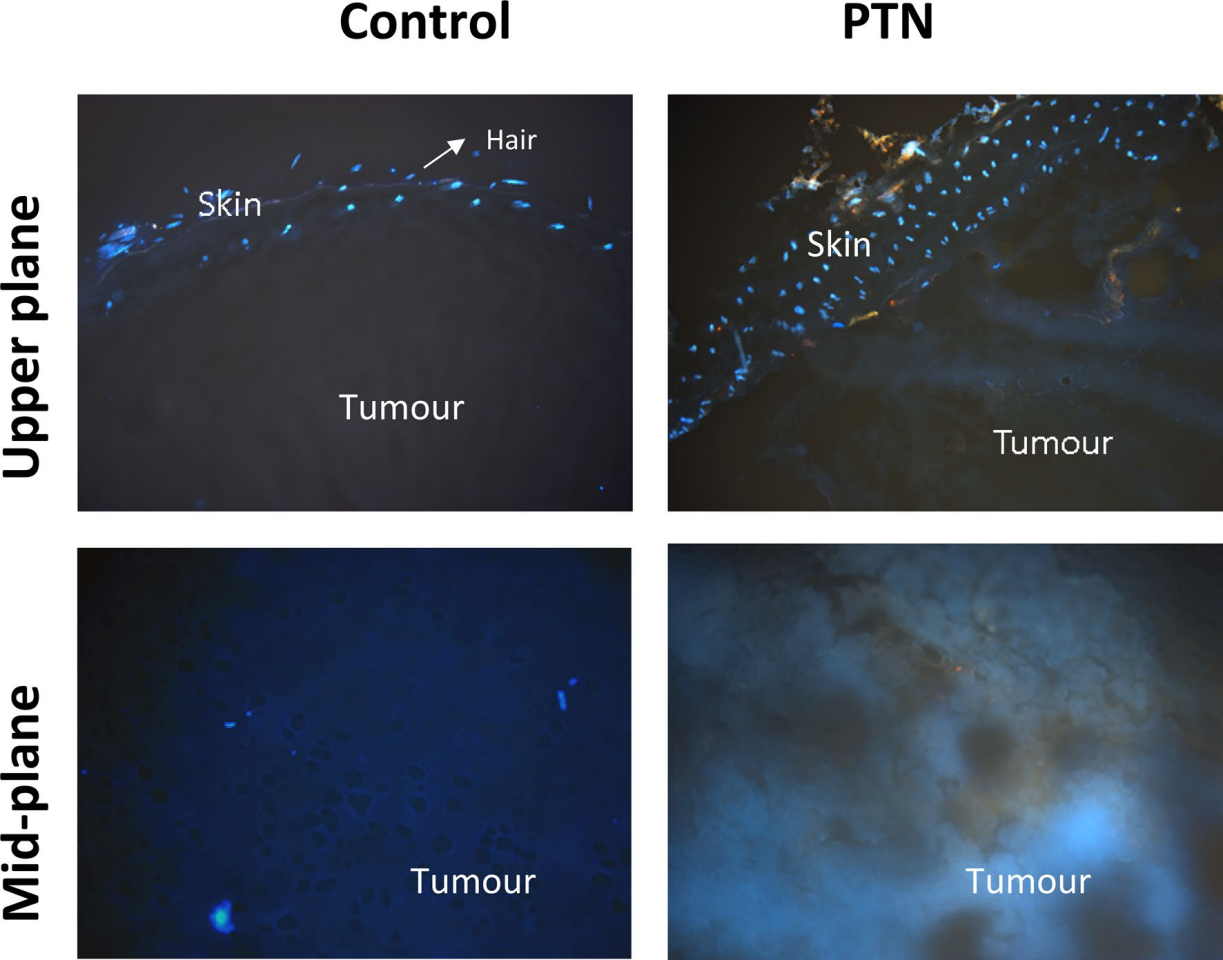
PTN penetration into the tumour. SOT was topically applied with 1.96 mg of PTN in DMSO. After 2 h, tumours were excised, and fluorescence of fresh sections was visualized by fluorescence microscopy. The basal superficial section of the skin (upper plane) and the deeper region (mid-plane) were documented at × 10. Controls treated with DMSO were included.

After topical application of PTN on the skin over the tumour, a characteristic orange fluorescence was noticed in the skin and tumour structures. Retention of the molecule in the stratum corneum and also penetration into the inner layers of the tumour up to a deep of approximately 5 mm was noticed. Control tissues treated with vehicle exhibited blue autofluorescence (Fig. 5).

**Figure 6:**
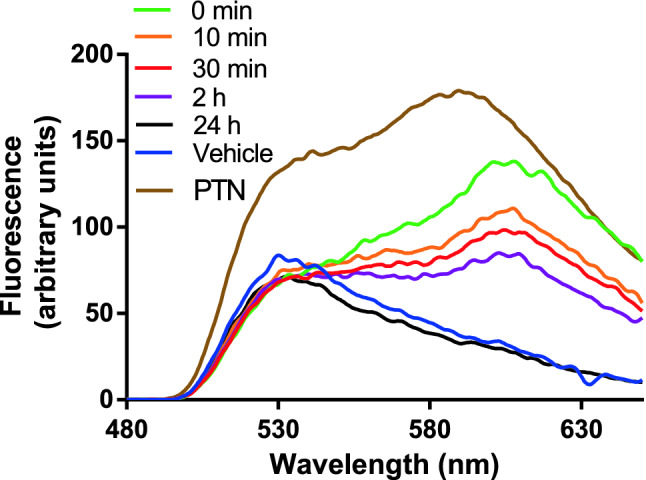
Kinetics of PTN penetration through the skin overlying the tumour. After topical application of PTN (1.96 mg in DMSO) on the skin over the tumour, fluorescence spectra of the AQ were recorded at the skin surface at different time points utilizing an optic fiber coupled to a spectrofluorometer. The decrease of superficial fluorescence accounts for the penetration of the molecule to inner tissue. Controls treated with DMSO (vehicle) and a fluorescence spectrum of PTN in DMSO (PTN) are included. Representative spectra are shown.

After topical application of PTN on the skin overlying the tumour tissue, superficial fluorescence was followed at different time points (Fig. 6). The PTN signal decreased after a few minutes after treatment, suggesting penetration of the molecule into the deeper layers of the skin. After 24 h of application, the fluorescence signal resembled the spectra of controls treated with the vehicle. Considering these data, we set the time point to perform PDT at 2 h after PTN application, time at which fluorescence decreased significantly at the skin surface and was located in the deep layers of tumour tissue.

**Figure 7:**
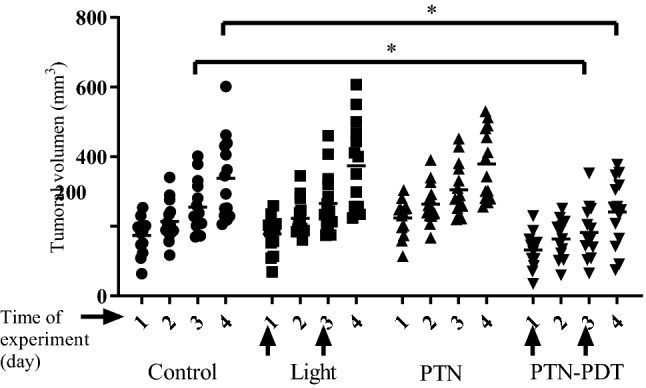
Delayed tumour growth after PTN-PDT. A DMSO lotion containing 1.96 mg of PTN was applied on the skin over the tumour and after 2 h the area was illuminated. This PTN-PDT treatment was repeated on days 1 and 3 of the experiment (arrows). Controls received vehicle treatment. Illumination and PTN-treated controls were also included. Tumour volume was followed by caliper measurement. *p < 0.05 control vs. PTN- PDT, two-way ANOVA, followed by Sidak's multiple comparisons test.

PDT employing blue light illumination was applied immediately after topical application of PTN on the skin over the subcutaneously implanted LM2 tumour, and tumour growth was followed by caliper measurement (Fig. 7). A single light dose at day 1 of the experiment induced a delay in tumour growth at day 3 of the experiment (p = 0.0048 and 0.004 respectively, as compared to the non-treated control, ANOVA followed by Sidak’s multiple comparisons). However, the delay was not maintained in time and therefore, we administered a second PTN-PDT boost on day 3. Under these conditions, the tumour growth was significantly decreased on day 4 of the experiment as well (p = 0.004 respectively, as compared to the non-treated control). The mean size for the control group at day 3 was 255.1 mm^3^ (range 169.4–401.8), whereas for PTN-PDT the mean was 182.1 mm^3^ (range 65.4–352.0). On day 4 of the experiment, the mean size for the control group was 337.7 mm^3^ (range 205.6–601.9), and for PTN-PDT, it was 240.8 mm^3^ (range 74.0–376.8). Therefore, considering the mean of tumour size of both the control and PDT treatments, the delay of tumour growth was 28% on average on days 3 and 4 of the experiment. From day 5 onwards, there were no significant differences between control and PTN-PDT-treated groups (data not shown). Controls of light and PTN treatment alone did not significantly affect tumour growth.

**Figure 8:**
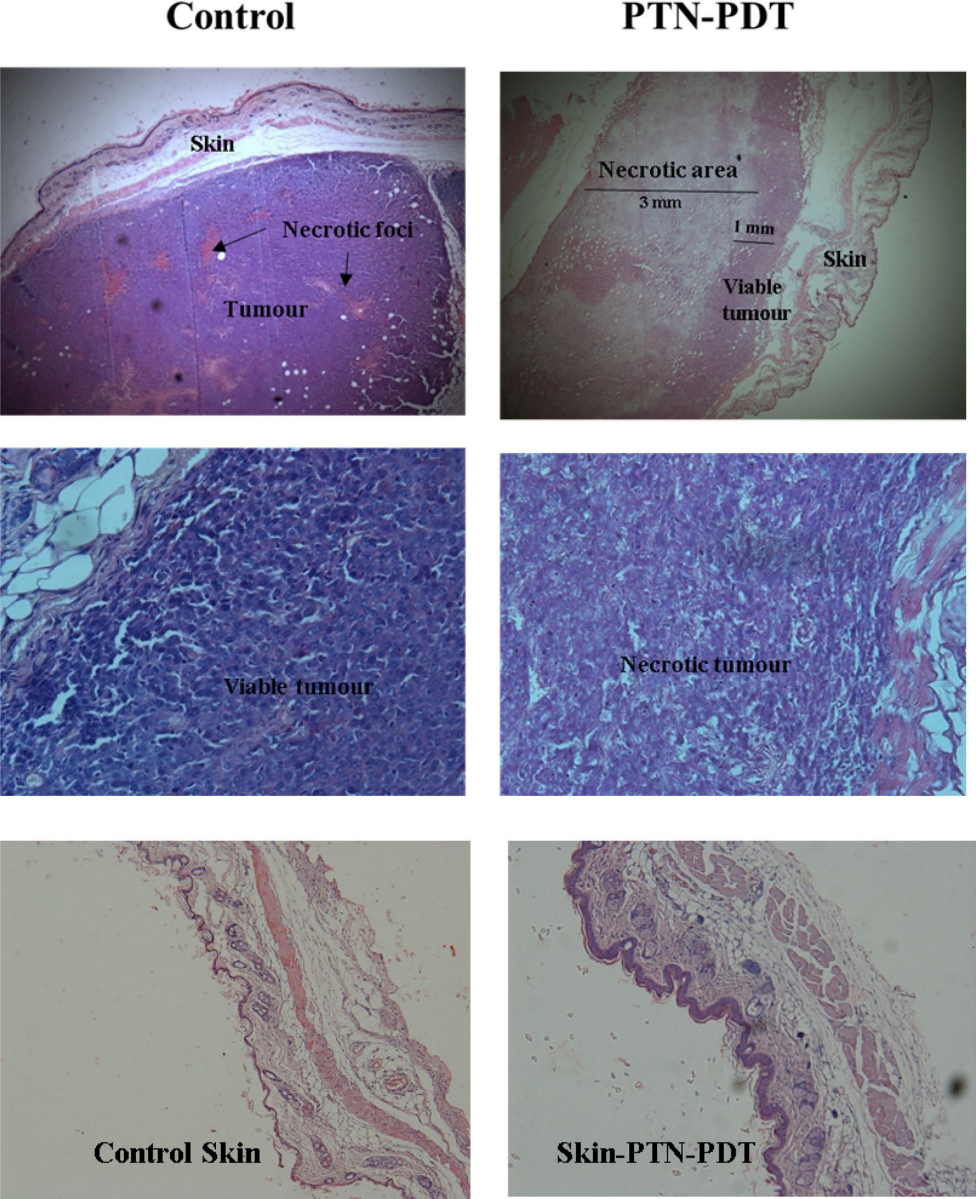
Hematoxylin and eosin-stained images of skin and adjacent tumours and normal skins treated with topical PTN-PDT. PTN-PDT treated tumours were excised 24 h after the second PDT dose (day 4 of the experiment). Controls of tumours treated with vehicle were included. Sections were photographed using the × 4 (upper panel) and × 40 (middle panel) objectives. Lower panel represents normal skins excised 24 h after the second PDT dose and non-treated controls (× 10).

Control tumours topically treated with vehicle exhibit random small foci of spontaneous necrosis. However, massive tumour necrosis was observed after PTN-PDT treatment in the deep area of the tumour up to 4 mm of depth. Intriguingly, a superficial area of viable tumour and quite conserved intact skin was evidenced in the 1 mm superficial area (Fig. 8). The histological damage observed in the tumours treated with PTN-PDT correlates with PTN penetration into the inner layers of the tumour (Fig. 5).

**Figure 9:**
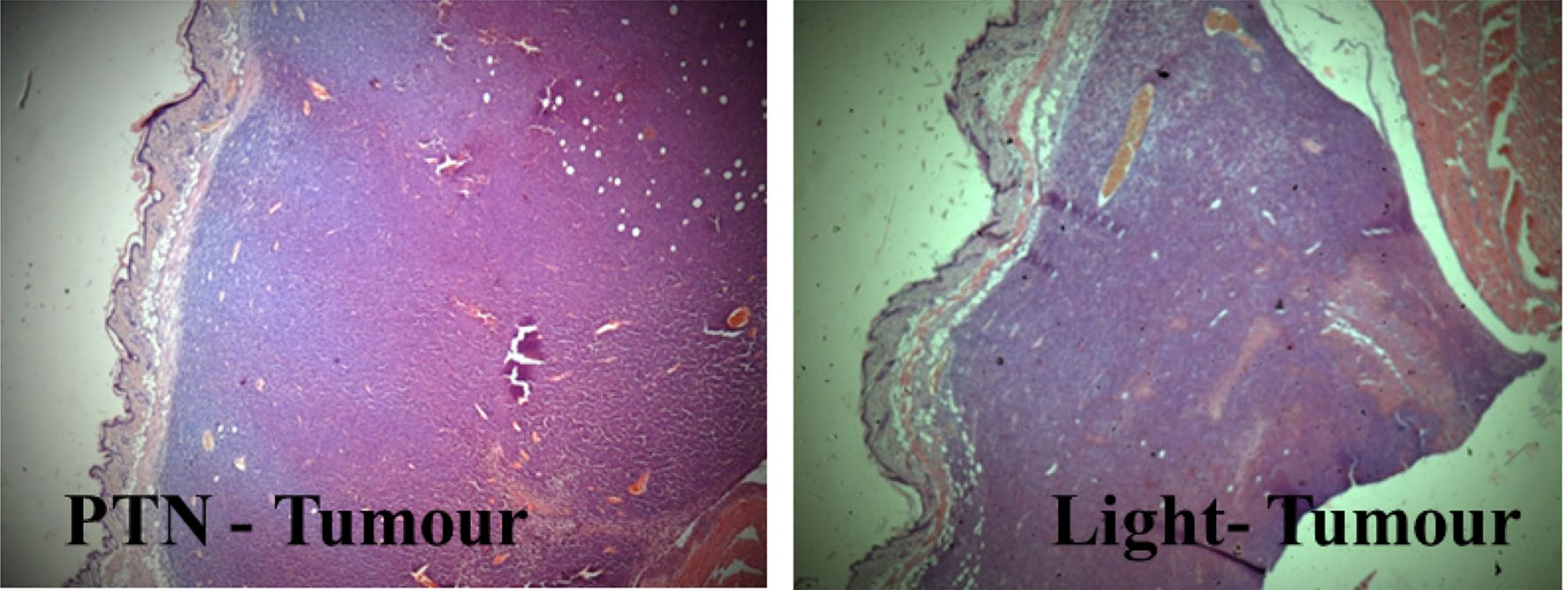
Control tumours treated with PTN or light alone. Tumours treated twice with PTN and non-illuminated (PTN-Tumour) or illuminated twice previous application of vehicle (Light-Tumour); mice were sacrificed and tissues excised 24 h after the last dose. Sections were photographed using the × 4 objective.

Skin treated with PTN-PDT exhibited slight changes such as dermal acanthosis, dermal fibrosis, and hypertrophy of the muscular layer of the skin. Control tumours treated with PTN or light alone were not affected by the treatments (Fig. 9).

## Discussion

Some intercalating AQs have been employed systemically for cancer treatment^11^, however to the best of our knowledge there are no reports of AQs employed in topical use for superficial tumours. Regarding other uses, natural AQs such as emodin, which is an AQ derivative isolated from the roots of *Rheum officinale* Baill, was topically administrated to rats in a hydrogel containing 2% carbopol for the treatment of skin wounds^33^. Moreover, the fact that PTN showed an increased in vitro permeation through pig skin as compared with emodin (48-fold), suggests the feasibility of this AQ as a promising molecule for dermal use^34^.

AQs, including PTN, act inhibiting tumour growth and metastasis, inducing apoptosis and cancer cell metabolism, re-programmation and cell cycle arrest, regulating cell signalling pathways, and improving the effectivity of other chemotherapeutics in drug-resistant cells^35^. Whereas some authors demonstrated that PTN was significantly cytotoxic in the dark when it was used at concentrations from 50 μM onwards after 72 h exposure, with a low percentage of apoptotic cells^36^; other authors demonstrated that lower exposure time to in vitro growing cells induced no significant effect on the inhibition of cancer cell proliferation (35 µM for 16 h^37^, and 40 and 80 µM for 12 h)^38^.

In our work, we aimed to unveil the phototoxic potential of PTN in tumour cells and therefore, we employed sub-toxic concentrations of PTN and low time exposure. We found that 1 h incubation of LM2 cells with 30 µM PTN induced less than 30% of cell death. Under these conditions, a light dose of 1.78 J/cm^2^ induced 50% of cell death, whereas 4.5 J/cm^2^ induced the maximal cell death achieved. Under the conditions of sub-lethal PTN cell killing, we performed an in vitro migration assay, and we found that PTN per se impaired cell migration upon illumination, and that the migration index was decreased twice. Similar results were found by Han et al.^16^ employing PTN at non-toxic concentrations (2.5 and 5 µM) in human colorectal cancer cells. Under those conditions, they found diminished cell adhesion, migration and invasion and epithelial–mesenchymal transition, thus inhibiting the in vitro metastatic potential by down regulation of the transcription factor SOX2. PTN also increased per se ROS production and remarkably, the ROS inhibitor *N*-acetylcysteine, suppressed the antimetastatic potential of PTN.

In the present work, we found that PTN exhibit in vitro cytoplasmic localization in LM2 cells, whereas no PTN fluorescence was found in the nucleus. The orange-green fluorescence observed upon UV excitation is ascribed to two PTN emission species. Similarly, upon excitation with 355 nm of an OH-anthraquinone solution dissolved in chloroform, two species (keto and enol) with maxima emission peaks of 440 and 590 nm were observed^39^.

PTN has a pKa1 value of 3.9 and pKa2 of 9.8, the last corresponding to the monoanionic form dissociation^31^. Therefore, PTN is monoanionic at physiological pH and considering its hydrophobicity, a cytoplasmic localization was expected. Some anthracycline derivatives bearing the anthraquinone ring do not locate in the nucleus but in lysosomes^40^, and the granular fluorescence of PTN could also agree with a lysosomal localization. On the other hand, other natural aglycone AQs such as rubiadin and soranjidiol exhibited fluorescence mainly in the cytoplasm and in the nucleus^20^.

According to our results, PTN penetrates at least up to 5 mm in this model of subcutaneously implanted tumours. It has to be remarked that the mammary implanted tumour disrupts the skin, making it more permeable for topically applied drugs. In line with PTN penetration, massive tumour necrosis up to a depth of 4 mm was observed in PTN-PDT treated tumours. Although blue light penetration into normal skin has been reported to be around 1 mm^41^, it had been used successfully in the treatment of skin cancers such as human basal cell carcinomas^42^ and melanoma mice lesions of 100–120 mm^3^^43^. In addition, a bystander effect that may propagate the cellular death triggered by the initial photoreaction may be also occurring^44^.

We were surprised to find that the upper layers (up to 1 mm) of the tumour remain viable after the two doses of PTN-PDT. In addition, both skins overlying tumours and normal skins topically treated with PTN-PDT did not show major signs of photodamage. We hypothesize that this fact may be explained by the vehicle employed for PTN solubilization. After PTN is dissolved in DMSO, upon topical administration, the molecule is likely to aggregate in contact with the skin and tumour milieu, and even form small crystals visible under fluorescence microscopic observation, similarly to the tiny extracellular crystals formed in the top layer of the lichen cortex^45^. It has been reported that the intracellular self-aggregation of PTN in acidic and neutral solutions avoids its photophysical and photobiological activities by quenching both singlet and triplet excited states^24^. This aggregated form of PTN is not photoactive, but in the deeper tumour layers, the concentration of PTN is lower and thus no aggregation processes take place. Moreover, acidic extracellular pH of tumours may be affecting PTN aggregation, since PTN aggregates at pH aqueous medium lower than 7^31^. Therefore, the vehicle employed for PTN solubilization may be crucial in the outcome of PDT and the depth of the photodamaged tissue.

The photosensitizing AQs of *Heterophyllaea pustulata* Hook. f. (Rubiaceae) including soranjidiol, soranjidiol 1-methyl ether, rubiadin, rubiadin 1-methyl ether, damnacanthal, damnacanthol, heterophylline, pustuline and the bianthraquinone (*S*)-5,5-bisoranjidiol^46,47^ induces dermal lesions in sheep after ingestion of the plant and exposure to sunshine. The affected skin showed diffuse necrosis of the entire epidermis and superficial dermis, even up to the depth of the apocrine sweat glands, whereas sebaceous glands still appeared viable. However, in the present work, we found that PTN induces minimal damage to normal skin upon topical application of PTN and illumination with two doses of blue light.

A single PTN-PDT dose was not enough to induce tumour delay or impaired growth. However, after two PTN-PDT doses separated by 1 day, the tumour growth was around 28% delayed at days 3 and 4 of the experiment, as compared to the non-treated control and the PTN and light-treated alone, thus accounting for a sustained growth delay for 2 days.

Breast cancer cutaneous metastases are incurable and the treatment is directed towards local control with surgical excision, radiation, and chemotherapy^48^. Therefore, considering that the mean human epidermal thickness ranges from 56.6 to 81.5 μm^49^, patients at early stages of the disease, without epidermal invasion, could benefit from PTN-PDT topical treatment in combination with other established therapies.

These preliminary findings encourage further studies of natural AQs administered in different vehicles, for topical treatment of cutaneous malignancies.

## Data Availability

The datasets generated during and/or analysed during the current study are available from the corresponding author on reasonable request.

## Abbreviations

AQ/Aqs: Anthraquinone/s
bf: Barrier filter
dm: Dichroic mirror
DMSO: Dimethyl sulfoxide
excf: Excitation filter
FBS: Fetal bovine serum
h: Height
l: Longer
MI: Migration index
MTT: 3-[4,5-Dimethylthiazol-2-yl]-2,5-diphenyl-tetrazolium bromide
PBS: Phosphate buffer saline
PDT: Photodynamic therapy
PS/PSs: Photosensitizer/s
PTN: Parietin
PTN-PDT: Photodynamic therapy mediated by parietin
SOT/SOTs: Skin/s overlying the tumour
w: Shorter
